# Extracellular vesicles from Distinct Strains Modulate Phagocyte Function and Promote Fungal Persistence

**DOI:** 10.1021/acsinfecdis.5c00378

**Published:** 2025-07-14

**Authors:** Taiane N. Souza, Alessandro F. Valdez, Ana Claudia G. Zimbres, Bianca A. G. Sena, Flavia C. G. Reis, Marcio L. Rodrigues, Daniel Zamith-Miranda, Allan J. Guimarães, Alessandra A. Filardy, Joshua D. Nosanchuk, Leonardo Nimrichter

**Affiliations:** † Instituto de Microbiologia Paulo de Góes, 28125 Universidade Federal do Rio de Janeiro, Rio de Janeiro 21941-902, Brazil; ‡ Division of Infectious Diseases, Department of Medicine, 2006 Albert Einstein College of Medicine, Bronx, New York, New York 10461, United States; Centro de Desenvolvimento Tecnológico em Saúde (CDTS ), Fundação Oswaldo Cruz, Rio de Janeiro 21941-902, Brazil; ∥ Instituto Carlos Chagas, Fundação Oswaldo Cruz (Fiocruz), Curitiba 81310-020, Brazil; ⊥ Department of Microbiology and Immunology, Albert Einstein College of Medicine, Bronx, New York, New York 10461, United States; # Departamento de Microbiologia e Parasitologia—MIP, Instituto Biomédico, Universidade Federal Fluminense, Rio de Janeiro 24210-130, Brazil; ¶ Rede Micologia RJ—Fundação de Amparo à Pesquisa do Estado do Rio de Janeiro (FAPERJ), Rio de Janeiro 28495-000, Brazil; ∇ National Institute of Science and Technology (INCT) in Human Pathogenic Fungi, São Paulo 14040-903, Brazil

**Keywords:** *Histoplasma capsulatum*, extracellular vesicles, innate immunity, virulence

## Abstract

Fungal extracellular vesicles (EVs) are lipid-bilayer compartments that transport a wide range of molecules, including proteins, polysaccharides, pigments, small metabolites, lipids, and RNA. In fungal pathogens, EVs harbor virulence factors as well as antigenic determinants that modulate the host immune response. In this work, we investigated the modulatory effects of EVs released by two phenotypically and genotypically distinct strains of (G-217B and G-184A) on bone marrow-derived macrophages (BMDMs) and bone marrow-derived dendritic cells (BMDCs). Both host cells internalized EVs, which appeared to elicit distinct functional responses. Treatment of BMDMs with EVs from either strain (EV_HcG‑184A_ and EV_HcG‑217B_) increased IL-6 production with no significant changes in IL-10 levels. In contrast, BMDCs exposed with both EVs exhibited elevated levels of IL-6 and IL-10. Although EV treatment led to increased inducible nitric oxide synthase expression in BMDMs, it did not stimulate NO production. Remarkably, both EVs reduced the metabolic activity of phagocytes. Overnight exposure to EV_HcG‑217B_ enhanced the phagocytosis of yeasts by BMDMs; however, the phagolysosomal fusion was not affected. Notably, in DCs, EV_HcG‑217B_ enhanced both the uptake and the viability of G-217B yeasts. Furthermore, incubation of with its respective EVs promoted fungal growth, suggesting a self-stimulatory mechanism that may contribute to fungal persistence within host cells. Taken together, our results support the idea that EVs are modulators of host–pathogen interaction, influencing phagocyte function and potentially contributing to fungal virulence.

## Graphical abstract

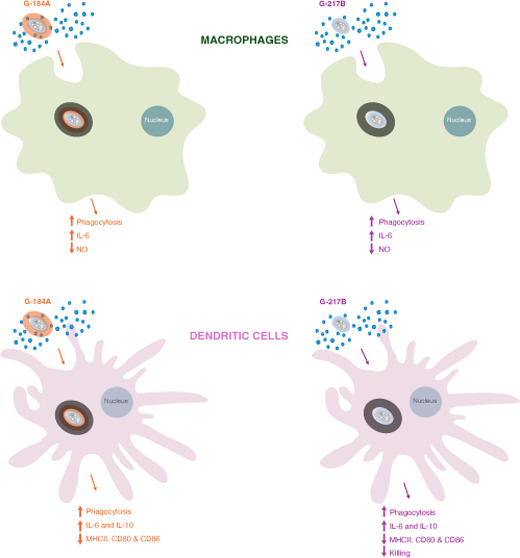

, the causative agent of histoplasmosis, is a dimorphic fungus prevalent in the Americas.^1,2^ Although infection typically remains localized in the lungs, the fungus can disseminate systemically, particularly in immunocompromised individuals, posing a significant risk of fatal disease.^3^ For instance, in Latin American countries, including Brazil, HIV-associated histoplasmosis remains the most clinically significant mycosis, resulting in more than 30% of deaths, despite the expansion of antiretroviral therapy.^1,4,5^

Primary histoplasmosis occurs upon inhalation of environmental microconidia and hyphae fragments. In the lungs, these structures reach the alveoli and convert into the yeast form, which represents the parasitic phase of . The yeast cell wall is the main interface with host cell receptors, and its composition is used to define chemotypes.^3,6^ The cell wall of chemotype II is primarily composed of chitin, β-glucans [including β-(1,3)- and β-(1,6)-linked glucans], with an outermost layer of α-(1,3)-glucan.^6,7^ In these strains, the α-1,3-glucan masks the underlying immunogenic β-1,3-glucan, preventing recognition by dectin-1, a C-type lectin receptor expressed by myeloid cells.^3,7,8^ In contrast, the α-1,3-glucan polysaccharide is absent in the chemotype I strains. These strains seem to evade immune detection by secreting β-glucanases, which trim exposed β-glucan, impairing recognition by host immune cells.^9,10^ In addition, the cell wall of chemotype I strains contains a higher proportion of chitin and a lower content of β-glucan compared to chemotype II; however, the implications of these compositional fluctuations on the fungal pathogenesis are unknown.^11^

The consequences of interactions with phagocytes following internalization can vary.^3^ For instance, within pulmonary resident macrophages, yeasts replicate and induce apoptosis, which turns out to be a key process for fungal dissemination.^12^ On the other hand, dendritic cells (DCs) can kill the yeasts, thereby helping to restrict fungal spread.^13^ However, the global mechanisms that lead to fungal clearance in a mammalian host are not completely understood.

Extracellular vesicles (EVs) are bilayered membrane compartments actively released by living cells.^14^ EVs function as carriers of diverse molecular contents that can be delivered to the extracellular space, modulating local and distant biological processes.^14,15^ In fungi, EV composition has been characterized across multiple species, revealing a complex cargo of structural and bioactive molecules, such as polysaccharides, glycans, proteins, lipids, nucleic acids, metabolites, and pigments.^14^ Previous studies have shown that fungal EVs, including those from , carry virulence factors and immunomodulatory molecules capable of influencing host immune responses and disease development.^15–17^ Notably, EVs cargo are qualitatively and quantitatively modified when yeasts of are incubated with antibodies targeting HSP60, a protein enriched in these compartments.^16,18^ These findings suggest that antibodies may regulate the biogenesis or cargo loading of fungal EVs, thereby affecting their biological properties. Moreover, macrophages incubated for 60 min with EVs from the G-217B strain showed a decrease in fungal internalization,^16^ demonstrating a direct effect of EVs on these host cells. Here, we investigated the immunomodulatory effect of EVs isolated from two virulent strains, chemotypes I (G-217B) and II (G-184A), in both macrophages and DCs. We demonstrated that EVs released by G-217B and G-184A strains are internalized by both phagocytes and affect their metabolism and activation, modulating cytokine production, such as IL-6 and IL-10. Differences were observed in the outcome of infected DCs according to the origin of the EVs. The composition of EVs and their direct effect on growth suggest that these compartments are key factors during histoplasmosis development.

## Results

### EV_HcG‑217B_ and EV_HcG‑184A_ Exhibit Different Sizes and Total Protein Levels

As diameter distribution and particle concentration are important steps in EV characterization,^19^ we used nanoparticle tracking analysis (NTA) to assess the sizes and concentration of EVs obtained from two different chemotypes of , G-217B (chemotype I) and G-184A (chemotype II) strains. EVs from G-217B strain (EV_HcG‑217B_) exhibited a range of 100 to 300 nm in diameter, whereas EVs from G-184A (EV_HcG‑184A_) showed a broader size distribution, ranging from 100 to 500 nm, displaying a predominant population of larger vesicles (Figure 1A–C). The EVs yield per yeast cell was similar for both strains (Figure 1D). The quantification of sterol and total protein content was also used as a parallel approach to characterize and quantify EVs collected from both strains. While sterol levels were comparable between EV_HcG‑217B_ and EV_HcG‑184A_, the latter displayed a higher protein concentration (Figure 1E,F). Given previous studies reporting the limitations of NTA in accurately measuring EVs with heterogeneous sizes,^20^ we chose to use sterol content as the quantification method for EVs in our subsequent experiments.

**Figure 1:**
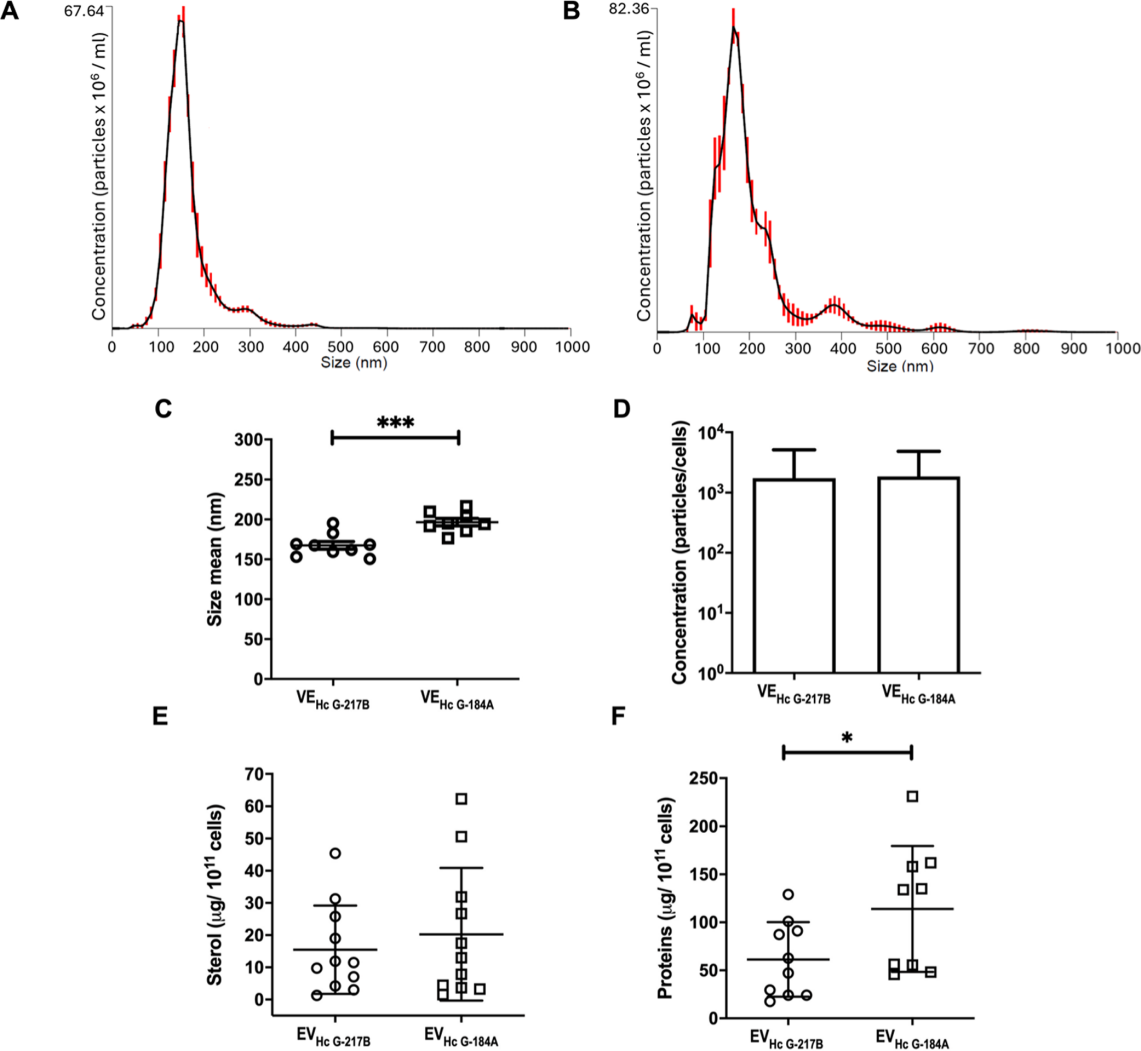
Dimensional profiling of EV_HcG‑217B_ and EV_HcG‑184A_. EVs were isolated from G-217B and G-184A strains and then subjected to NTA analysis of size and concentration. Representative NTA histograms of size distribution for (A) EV_HcG‑217B_ and (B) EV_HcG‑184A_ are shown. (C) Mean particle size analysis of EV_HcG‑217B_ and EV_HcG‑184A_. (D) EV_HcG‑217B_ and EV_HcG‑184A_ production were each normalized by the number of yeasts after 48 h in growth culture. Compositional analysis of (E) sterol and (F) protein in EV_HcG‑217B_ and EV_HcG‑184A_. Graphs show means ± SD from at least nine EV isolations. *p < 0.05; ***p < 0.001, significance was evaluated by Student’s *t*-test.

### EV_HcG‑217B_ and EV_HcG‑184A_ Are Internalized by the BMDMs and BMDCs

Distinct internalization patterns of Dil-stained EV_HcG‑217B_ and EV_HcG‑184A_ by BMDMs and BMDCs were observed. After 1 h of incubation, neither EV_HcG‑184A_ nor EV_HcG‑217B_ was detected inside BMDMs, while a small number of fluorescent cells were observed in BMDCs (Figure 2A,B). Following overnight incubation, both EV_HcG‑217B_ and EV_HcG‑184A_ were internalized by BMDM and BMDC with a more pronounced signal intensity (Figure 2A,B). These results suggest that EVs from are internalized by BMDMs and BMDCs with distinct kinetics, which may differ in impacting cell activation and immune responses.

**Figure 2:**
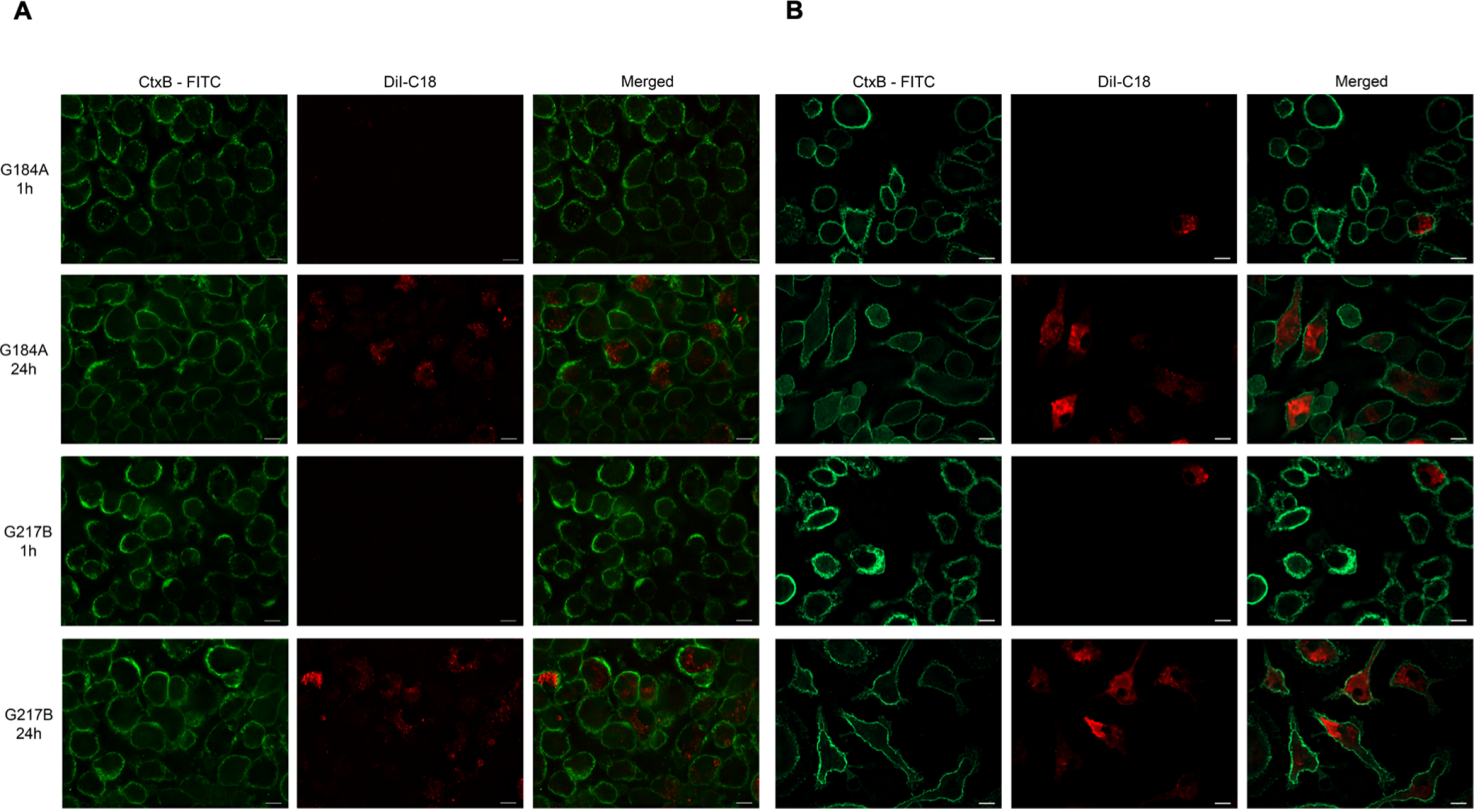
Internalization of EV_HcG‑217B_ and EV_HcG‑184A_ by BMDMs and BMDCs. Dil-stained EV_HcG‑217B_ or EV_HcG‑184A_ (red) was incubated with BMDMs or BMDCs for 1 and 24 h. The plasma membrane was stained with cholera toxin B subunit conjugated with FITC (green). Images of BMDMs (A) and BMDCs (B) were merged with the corresponding fluorescence. Scale bars, 10 μm.

### EVs Derived from Affect the Metabolic Activity of BMDMs and BMDCs without Affecting Their Viability

To investigate the direct effects of EV_HcG‑217B_ and EV_HcG‑184A_ on phagocyte functions, we initially assessed their cytotoxic effects in BMDMs and BMDCs by using the MTT assay. Both EV types induced a dose-dependent reduction in metabolic activity in both cells (Figure 3A,B). Despite this decrease, the morphology of EV-treated BMDMs remained comparable to that of untreated cells (data not shown). To further investigate the potential cytotoxicity, we assessed the lactate dehydrogenase (LDH) activity in EV-treated phagocytes. Our results showed that neither EV_HcG‑217B_ nor EV_HcG‑184A_ induced cell death (Figure 3C,D). Moreover, even at elevated concentrations, both EVs failed to trigger apoptosis or necrosis in BMDMs, as evidenced by the high percentage of annexin V-negative and PI-negative cells, comparable to that of the negative control (Figure S1). These results suggest that EVs did not cause damage to macrophages, although they do modulate the metabolic activity of host cells.

**Figure 3:**
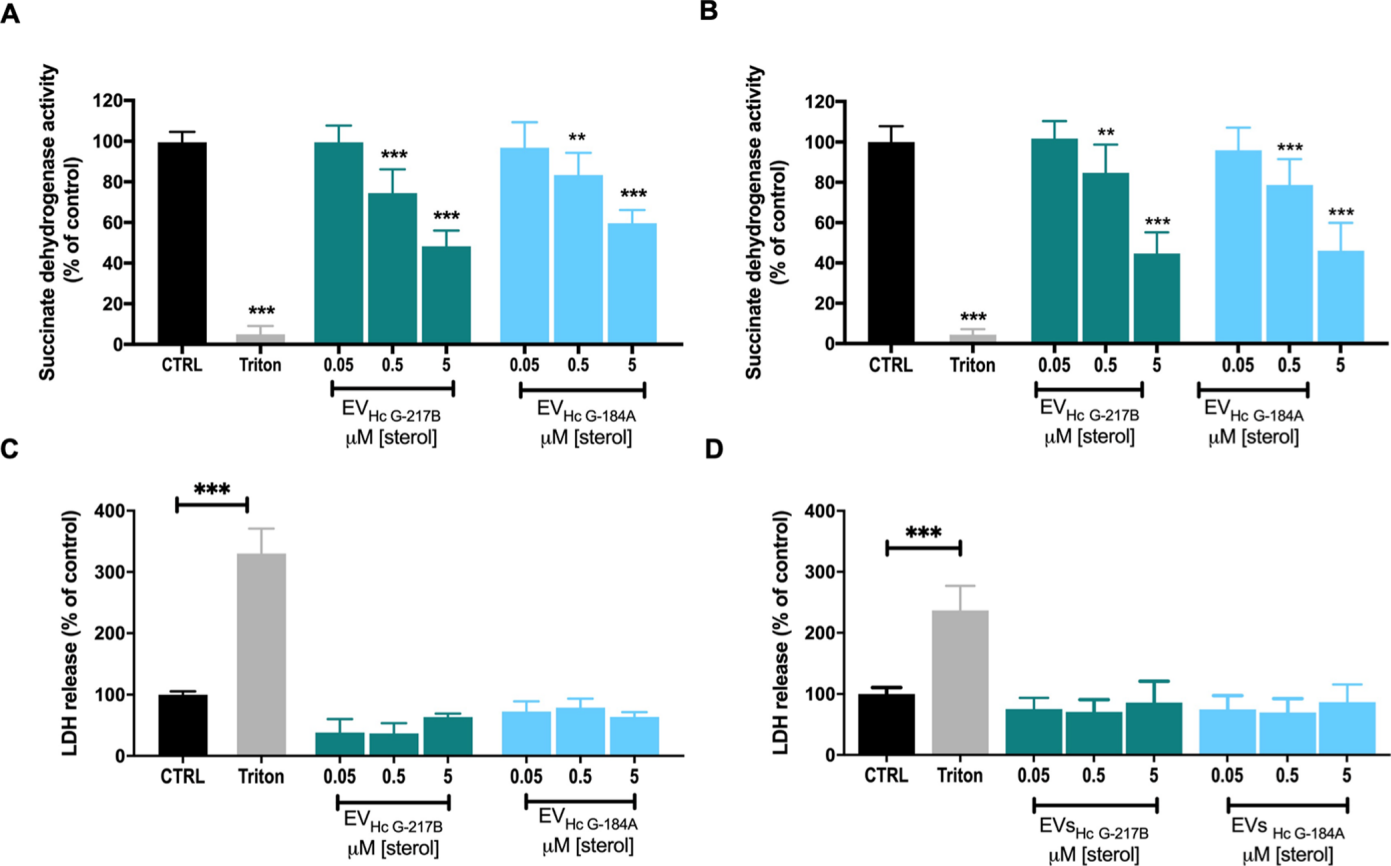
Viability of phagocytes treated with EV_HcG‑217B_ or EV_HcG‑184A_. BMDMs and BMDCs were stimulated overnight with different concentrations of EV_HcG‑217B_ or EV_HcG‑184A_. Cellular metabolism was evaluated by measuring the succinate dehydrogenase (SDH) activity of BMDMs (A) and BMDCs (B) using MTT. LDH activity was measured in the supernatant of BMDMs (C) and BMDCs (D). Lysis with Triton X-100 was used as a positive control for the MTT and LDH assays. Graphs show means ± SD from 3 independent experiments. **p < 0.01; ***p < 0.001, one-way ANOVA comparison to untreated cells followed by the Bonferroni correction.

###  EVs Induce a Proinflammatory Phenotype in BMDCs without Affecting Their Maturation

To better understand the effects of EVs on phagocyte activation, we examined the production of inflammatory mediators by the BMDMs and BMDCs. Our findings indicated that EVs from both strains induce a proinflammatory response in BMDMs, characterized by a dose-dependent increase in IL-6 levels following overnight stimulation, while IL-10 levels remain unaffected (Figure 4A,B). Furthermore, both EV_HcG‑217B_ and EV_HcG‑184A_ significantly increased inducible nitric oxide synthase (iNOS) expression levels in a dose-dependent manner (Figure 4C). However, nitric oxide (NO) production following treatment with EV_HcG‑217B_ or EV_HcG‑184A_ did not differ significantly from that of the untreated controls. Additionally, arginase-mediated hydrolysis of l-arginine to urea was not detected in BMDMs, suggesting that substrate availability for NO synthesis was not limited (Figure 4D,E).

**Figure 4:**
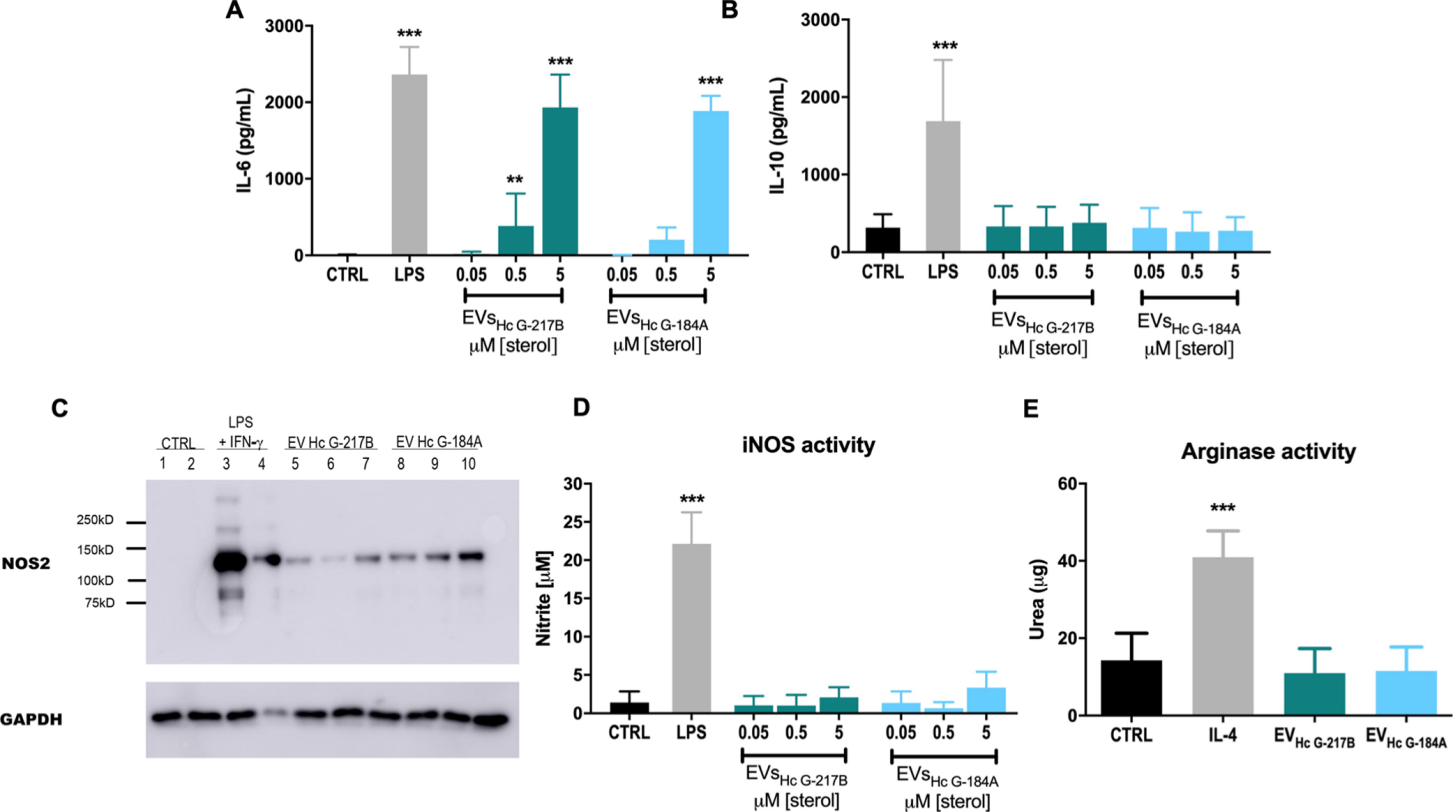
Inflammatory mediators and NO production by BMDMs stimulated with EV_HcG‑217B_ or EV_HcG‑184A_. BMDMs were incubated overnight with different concentrations of EV_HcG‑217B_ or EV_HcG‑184A_, and (A) IL-6 and (B) IL-10 concentrations in the supernatant were determined by capture ELISA. (C) iNOS content in cellular extracts was determined by Western blotting after treatment of BMDMs with 5 μM of EV_HcG‑217B_ or EV_HcG‑184A_. Treatment of BMDMs with IFN-γ (10 ng/mL) and LPS (500 ng/mL) was used as a positive control. (D) NO release was evaluated by measuring nitrite levels in cell supernatant through Griess reaction. (E) Arginase activity in cellular lysates was determined by measuring urea concentration. IL-4 (100 ng/mL) was used as a positive control to promote urease expression. Graphs show means ± SD from at least 3 independent experiments. **p < 0.01; ***p < 0.001, one-way ANOVA comparison to untreated cells followed by the Bonferroni correction.

Consistent with findings in BMDMs, EVs from both strains induced IL-6 production in BMDCs. Notably, BMDCs exhibited a higher sensitivity to EV_HcG‑217B_, with lower concentrations being sufficient to stimulate IL-6 production compared to BMDMs (Figure 5A). In contrast to BMDMs, IL-10 levels increased in BMDCs stimulated with the highest concentration of EVs from both strains (Figure 5B), but IL-12 levels remained unaltered (Figure 5C). We further assessed the effect of EVs on BMDC maturation by evaluating the levels of costimulatory molecules CD80, CD86, and MHC-II in CD11c^+^ cells. EVs derived from strain 11 (EV_Ca11_) were used as a positive control for maturation marker upregulation.^20^ In contrast to EV_Ca11_, EV_HcG‑217B_ or EV_HcG‑184A_ stimulation did not induce upregulation of CD80, CD86, or MHCII in BMDCs (Figure 5D,E).

**Figure 5:**
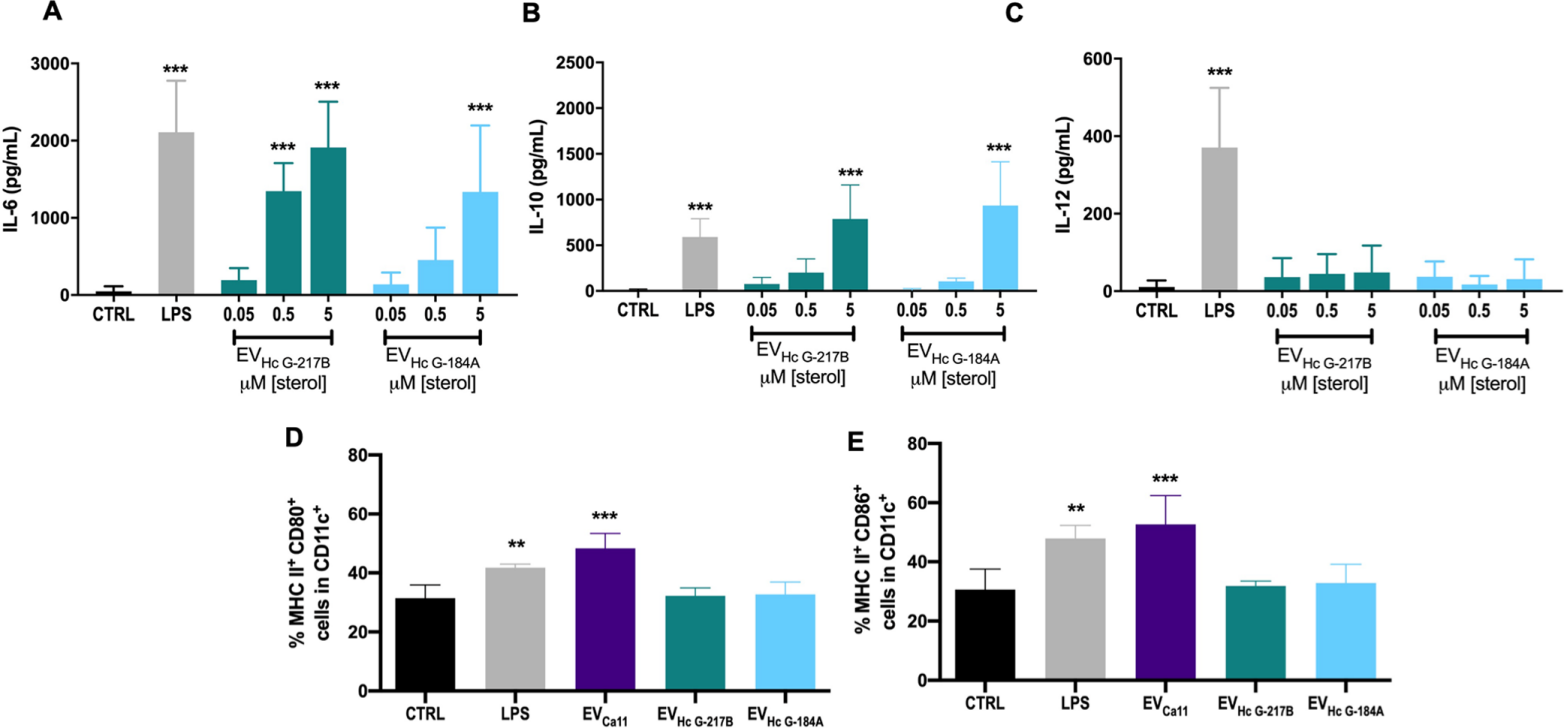
Inflammatory mediators and cell surface expression of CD80, CD86, and MHCII after treatment of BMDCs with EV_HcG‑217B_ or EV_HcG‑184A_. BMDCs were incubated overnight with EV_HcG‑217B_ or EV_HcG‑184A_, and (A) IL-6, (B) IL-10, and (C) IL-12 concentrations in the supernatant were determined by capture ELISA. Surface levels of CD86, CD80, and MHC-II were evaluated by flow cytometry. (D) Cell surface levels of MHC class II and CD80 in CD11c-positive cells. (E) Cell surface levels of MHC class II and CD86 in CD11c-positive cells. Graphs show means ± SD from 3 independent experiments. **p < 0.01; ***p < 0.001, one-way ANOVA comparison to untreated cells followed by the Bonferroni correction.

###  EVs Selectively Enhance the Phagocytosis of Yeast by BMDM and BMDC

Given the modulatory effects of EVs on BMDM and BMDC activation, we next investigated whether these EVs influence their phagocytic activity. We found that both BMDMs and BMDCs efficiently internalized strains G-217B and G-184A (Figure 6A,C). Notably, pretreatment with EV_HcG‑217B_ enhanced the uptake of the G-217B strain by both phagocytes. However, pretreatment with EV_HcG‑184_ increased the internalization of the G-184A strain in BMDCs but did not affect BMDMs (Figure 6A,C).

**Figure 6:**
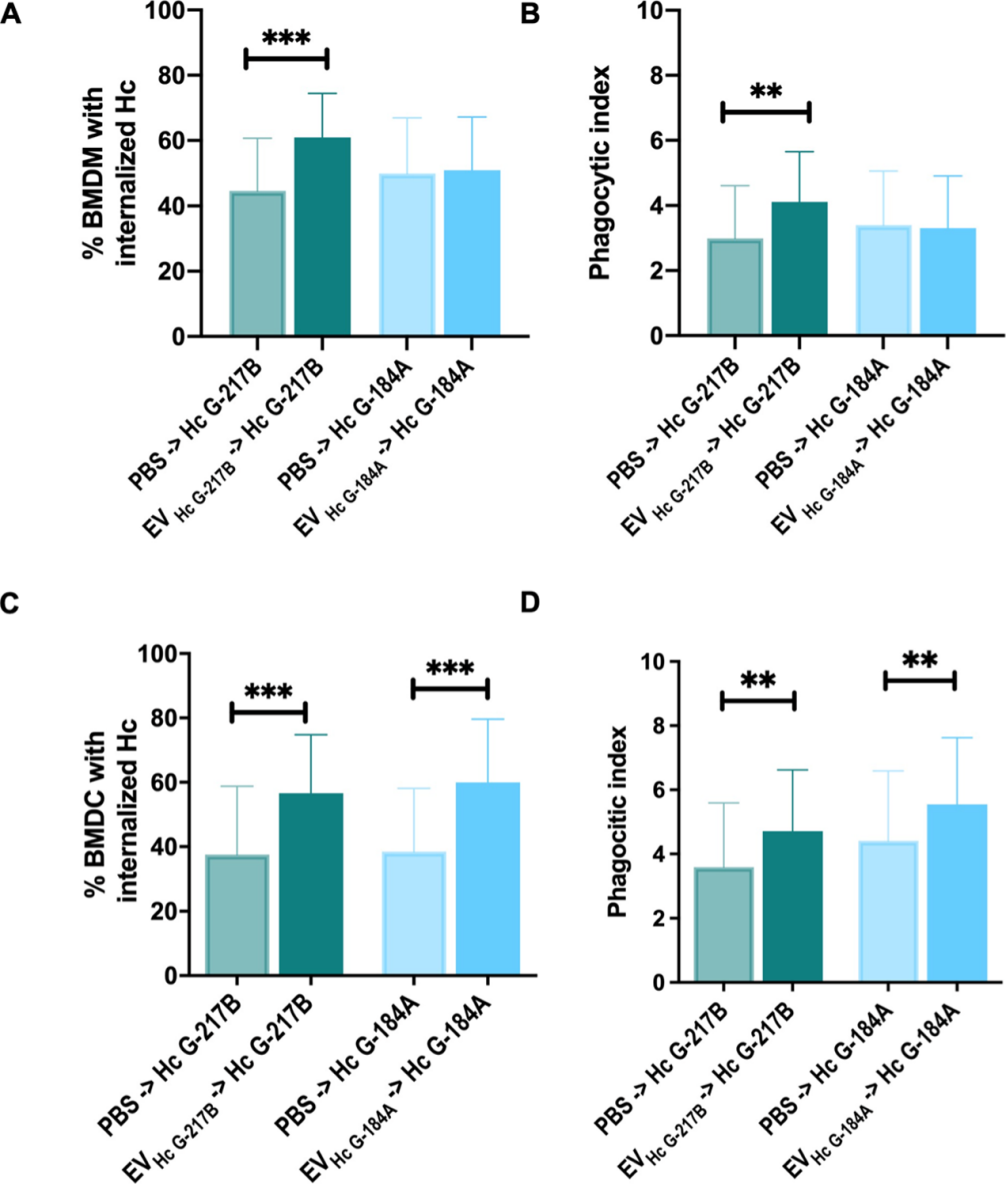
Phagocytosis of strains by BMDMs and BMDCs treated with EV_HcG‑217B_ or EV_HcG‑184A_. Phagocytes were treated overnight with EV_HcG‑217B_ or EV_HcG‑184A_ prior to the in vitro challenge with . Yeasts were prestained with NHS-rhodamine followed by coincubation with BMDMs for 1 h. Noninternalized fungal cells were counterstained with Uvitex 2B, and the internalization rates of yeast analyzed using fluorescence microscopy. (A) Number of BMDM with internalized yeasts. (B) Phagocytic index of BMDM. (C) Number of BMDC with internalized yeasts. (D) Phagocytic index of BMDC. Graphs show means ± SD from 3 independent experiments. **p < 0.01; ***p < 0.001, one-way ANOVA comparison to untreated cells followed by the Bonferroni correction.

We also assessed the average number of yeasts within phagocytes (phagocytic index) with and without EV pretreatment overnight. In BMDMs, a significant increase was observed only upon treatment with EV_HcG‑217B_ (Figure 6B). In contrast, BMDC pretreatment with either EV_HcG‑217B_ or EV_HcG‑184A_ increased the phagocytic index for yeasts (Figure 6D).

###  EVs Do Not Affect Phagosomal Acidification in Infected Macrophages

 yeasts employ a strategy of inhibiting phagolysosome fusion (PL-fusion) in both human and murine (RAW264.7) macrophages to evade immune-mediated killing.^21,22^ To assess whether EV pretreatment influences the fungicidal activity of BMDMs, we analyzed the PL-fusion using the acidotropic dye LysoTracker to quantify the acidification of BMDM phagosomes after ingestion of yeasts. As expected, heat-killed yeast, used as a positive control, significantly increased LysoTracker accumulation in BMDM phagosomes, with over 90% of phagosomes showing acidification (Figure 7A–C). In contrast, preincubation of macrophages with EV_HcG‑217B_ or EV_HcG‑184A_ had no effect on phagosome acidification in cells infected with live yeasts, indicating that EVs do not interfere with the PL-fusion process (Figure 7C). Collectively, our findings suggest that although EV_HcG‑217B_ can elicit an inflammatory response in macrophages, this activation is not sufficient to alter phagosomal acidification, a critical mechanism for macrophage protection against .

**Figure 7:**
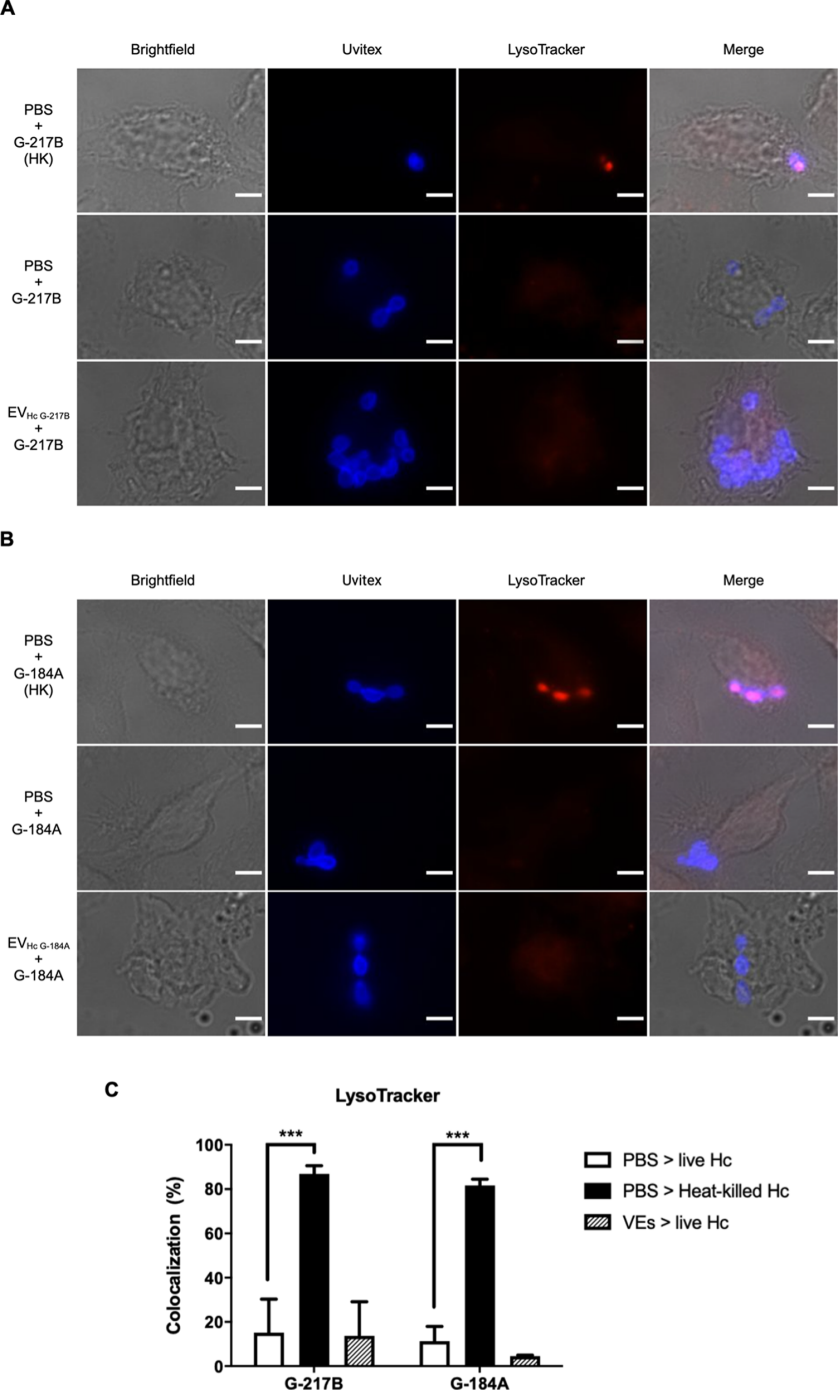
PL-fusion of strains by EV-treated BMDMs. BMDMs were treated overnight with EV_HcG‑217B_ or EV_HcG‑184A_ prior to in vitro challenge with . To evaluate PL-fusion, were prestained with Uvitex 2B and after phagocytosis, macrophages were incubated with the fluorescent acidotropic probe LysoTracker Red for 30 min. Fluorescence images of PL-fusion by colocalization of the LysoTracker (red) to Uvitex 2B-stained yeast (blue) for (A) G-217B and (B) G-184A are shown. (C) Percentage of colocalization. Graphs show means ± SD from 3 independent experiments. ***p < 0.001, one-way ANOVA comparison to untreated cells followed by the Bonferroni correction. Scale bars, 5 μm.

###  EVs Modulate the Yeast-Killing Activity of BMDC

Previous studies have demonstrated that PL-fusion proceeds normally in BMDCs following the uptake of yeasts.^13^ We then decided to evaluate whether pretreatment of BMDC with EVs would impact their antifungal activity. Pretreatment with EV_HcG‑217B_ enhanced the number of viable G-217B strain yeasts in BMDCs (Figure 8), whereas EV_HcG‑184A_ had no significant impact on the intracellular viability of G-184A yeasts (Figure 8). These observations raise the possibility that strain-specific EV components modulate immune cell function in different ways, potentially altering the effectiveness of the antifungal response.

**Figure 8:**
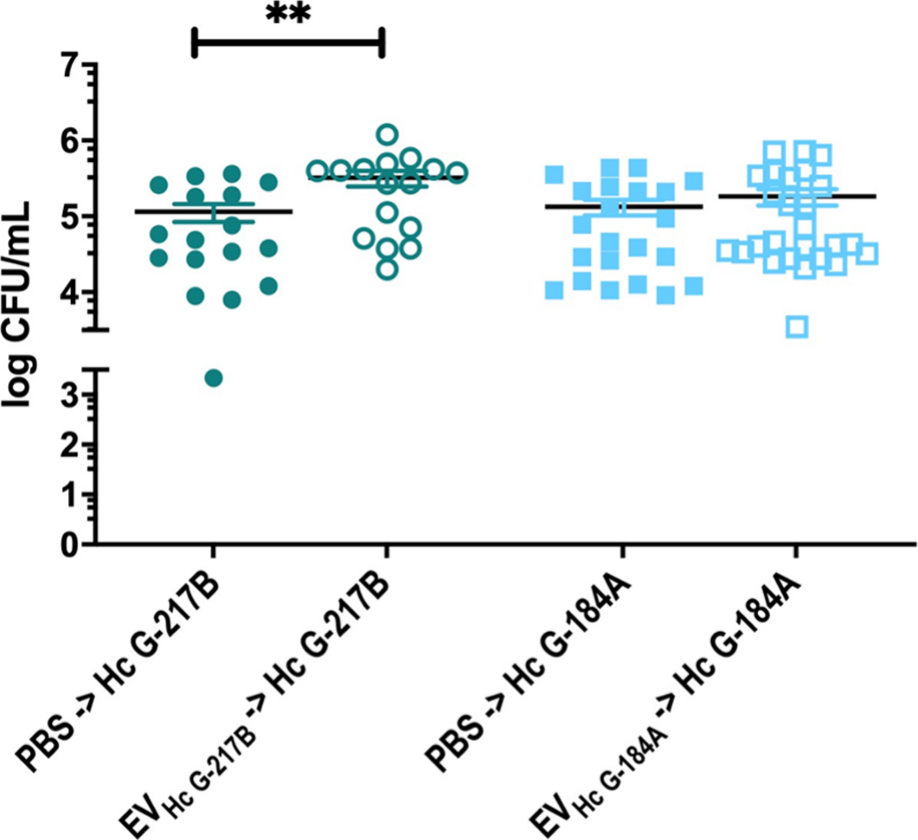
Antifungal activity of BMDCs treated with EV_HcG‑217B_ or EV_HcG‑184A_. The killing ability of BMDCs was evaluated after pretreatment of host cells with EV_HcG‑217B_ or EV_HcG‑184A_. Treated BMDCs were then infected with yeasts and incubated for 1 h. Nonadherent fungi were removed by washing with phosphate-buffered saline (PBS), and the systems were incubated for additional 2 h before cell lysis and plating on solid media. Yeasts viability was determined by colony forming units (CFU) counts per volume (mL). **p < 0.01, one-way ANOVA comparison to untreated cells followed by the Bonferroni correction.

### EVs from Exhibit Catalase Activity and Stimulate the Growth of Yeast Cells

To elucidate the role of EVs in the intracellular survival of different strains, we assessed catalase activity, an enzyme that is carried by EVs. This evaluation is particularly relevant, as functional catalases, especially catalase B, released by EVs have been associated with this survival capability in vitro, within polymorphonuclear leukocytes, and in vivo.^15,16,18^ We measured the catalase activity of EV_HcG‑217B_ and EV_HcG‑184A_ by monitoring the decrease of the OD at 240 nm using a spectrophotometer, with an initial OD of H_2_O_2_ set at approximately 0.2 (20 mM H_2_O_2_). No measurable H_2_O_2_ consumption occurred in the negative control (absence of the enzyme). However, significant H_2_O_2_ decomposition occurred when bovine catalase was added (positive control). Our results showed detectable catalase activity in EV_HcG‑217B_, as indicated by increased substrate consumption. Even so, no statistically significant differences in catalase activity were observed between the two EV types (Figure 9).

**Figure 9:**
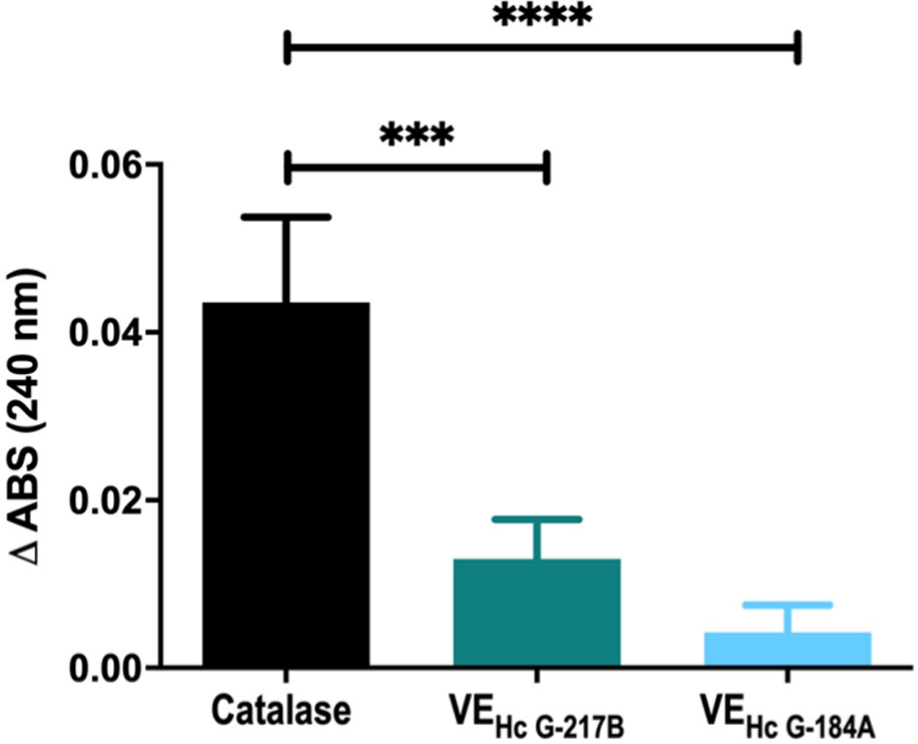
Catalase activity in EV_HcG‑217B_ and EV_HcG‑184A_. Enzymatic activity of catalase in EV_HcG‑217B_ and EV_HcG‑184A_ spectrophotometrically monitored at 240 nm. Graphs show means ± SD from 3 independent experiments. ***p < 0.001; ****p < 0.0001, one-way ANOVA comparison all the groups followed by the Bonferroni correction.

Finally, we investigated the direct effects of EV_HcG‑217B_ and EV_HcG‑184A_ on growth. Growth kinetics suggested that the addition of EVs to their respective strains (G-217B and G-184A) in vitro led to a marked increase in fungal growth (Figure 10).

**Figure 10:**
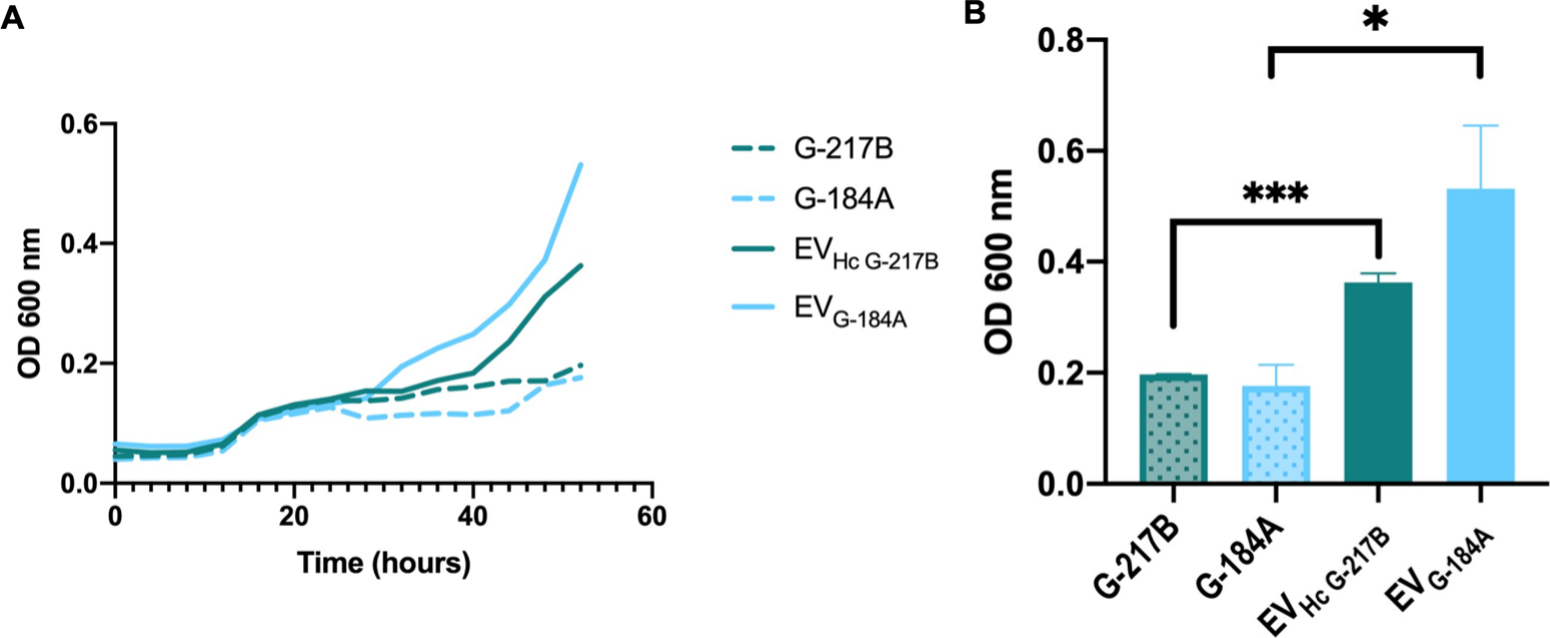
Direct effect of EV_HcG‑217B_ or EV_HcG‑184A_ on growing kinetics of the . (A) Growth curves were obtained after cultivation of G-217B and G-184A at 37 °C in the absence or presence of EV_HcG‑217B_ or EV_HcG‑184A_, respectively. The graph shows the mean of 3 independent experiments. (B) The OD measured after 52 h of G-217B and G-184A growing in the absence and presence of EVs. Graphs show means ± SD from 3 independent experiments. *p < 0.05; ***p < 0.001, comparison to untreated cells by Student’s *t*-test.

## Discussion

In this work, we investigated the effect of EVs released by two distinct strains of on BMDMs and BMDCs. The variations observed in EV size and protein-to-sterol content suggested variable cargo and, consequently, biological functions. This type of variability was also observed in EVs produced by different strains,^23^ suggesting that intraspecies EV diversity is higher than initially believed. We then hypothesized that EV_HcG‑217B_ and EV_HcG‑184A_ would differentially affect phagocyte functions. We first investigated the internalization of EVs by both phagocytes. Remarkably, the time required for EV internalization was notably longer than previously reported for EVs from other fungal pathogens.^24–26^ For instance, EVs are rapidly recognized and internalized by macrophages and DCs within minutes.^25^ In contrast, EVs showed no association with phagocytes within 5 and 30 min (data not shown) and remained largely undetectable in macrophages even after 1 h of incubation. It is conceivable that components carried by EVs may mask their immediate recognition. These results suggested that EVs from may contain components distinct from those found in EVs of other fungal species or that their structural organization may differ. In recent work, we demonstrated that EVs contain mannoproteins, β-1,3-glucans, and chitin oligomers. According to these results, the mannoproteins were recognized mainly by TLR4,^27^ which is consistent with findings by Rizzo and colleagues, who observed a strong reactivity of EVs from to concanavalin A and the presence of fiber-like structures resembling mannoproteins.^28^ We hypothesized that these mannoproteins may mask the other EV components in EVs, limiting their recognition by host cells. Although the presence of specific cell wall components in EVs has not been investigated, our findings indicate that if present, these molecules are likely not exposed on the vesicle surface. A masking mechanism similar to that observed on yeasts of may also occur on EVs. For instance, the presence of α-1,3-glucan in EVs from EV_HcG‑184A_ could reduce the initial binding to phagocytes. In parallel, EV_HcG‑217B_ might limit β-1,3 glucan exposure via the activity of glucanases, previously identified in EVs.^15–17^ Another factor that may modulate EV interaction with macrophages is Hsp60, a protein enriched in both cell wall and its EVs, and Hsp60 is known to interact with CD18, a component of CR3 on macrophages.^29,30^ Additionally, differences in chitin content between strains G-217B and G-184A may influence the presence of chitin-like molecules in their respective EVs. Further studies are necessary to identify the specific molecules involved in PRR engagement and to elucidate why the association of EVs with phagocytes is markedly slower compared to that of EVs from other fungal species. In EVs, these studies seem to be more challenging considering the structural variability in the distinct chemotypes.

Despite such structural diversity, many fungal EVs carry conserved immunostimulatory compounds that promote a proinflammatory response in macrophages and DCs.^26^ We first investigated the cytotoxicity of EVs in BMDMs and BMDCs. Our findings showed a reduction in SDH activity in both cell types. However, this metabolic impairment was not associated with cell death and may instead reflect M1-like polarization in BMDMs, as demonstrated by Van den Bossche and colleagues.^31^ According to these authors, this metabolic shift is linked to the inability of murine M1 macrophages to repolarize toward an M2 phenotype. Supporting this interpretation, both EVs increased IL-6 and iNOS expression in BMDMs but had no effect on IL-10 or arginase activity, suggesting a shift toward a proinflammatory profile, similar to what has been described for EVs from and .^32,33^ However, EV-treated BMDMs were not able to produce detectable amounts of NO, the effector molecule known to inhibit the SDH activity. It is possible that EVs from directly interfere with NO production or stability, or that other physiological mechanisms, such as rapid NO scavenging or compartmentalization, may have hindered its detection under our experimental conditions, but further studies are needed to confirm these hypotheses. Another raised possibility is a deficiency in essential enzymatic cofactors, such as tetrahydrobiopterin (BH4) and flavin mononucleotide, which are required for iNOS activity.^34^ Increased levels of ROS could oxidize cofactors, but our results did not show differences in ROS production when compared to control conditions (data not shown). It is important to mention that reduced ROS was previously observed when macrophages were incubated with EV_HcG‑217B_ for short periods (1 h), but in our study, host cells were stimulated overnight.^16^ Although we still do not understand how the NO is impaired, our results suggest that EVs might be implicated in fungal virulence.

The absence of NO production is just one aspect indicating that EVs might compromise the phagocyte response. EVs from both strains also failed to induce an increase in MHC class II, CD86, and CD80 levels on BMDCs. Furthermore, BMDCs treated with EV_HcG‑217B_ and EV_HcG‑184A_ produced IL-10 and IL-6 but not IL-12, a critical cytokine for the assembly of a Th1 polarization.^35^ A similar pattern has been observed with EVs from , particularly those derived from more virulent strains isolated from murine infected tissues.^36^ These EVs did not enhance the expression of MHCII and costimulatory molecules but induced the production of IL-6 and IL-10, alongside lower levels of the Th1-associated cytokines such as TNF-α and GM-CSF.^36^ Consequently, these DCs induced a high frequency of regulatory T cells (Tregs).^36^ These findings are consistent with our observations and support the idea of a shared EV-mediated virulence strategy among dimorphic fungi during infection. In contrast, and confirming our previous work, EVs from increased the number of activated cells (MHCII^+^CD86^+^) at levels, even higher than those induced by LPS, and stimulated a robust IL-12p40 production.^25^

In a previous study, we demonstrated that the pretreatment of macrophages with EV_HcG‑217B_ for 1 h partially inhibited the phagocytosis of yeasts.^16^ Since 1 h is the minimal incubation time required for EV internalization, we speculated that this reduction might result from competitive binding of EVs to the macrophage receptors. In contrast, here, we observed that overnight incubation with EV_HcG‑217B_ significantly enhanced phagocytosis by both phagocytes. A similar effect was observed with EVHc_G‑184A_, although only in BMDCs.

The consequences of enhanced phagocytosis may vary depending on the phagocyte type and its activation status. yeasts are known to survive within macrophages by inhibiting PL-fusion or maintaining the phagolysosome pH to 6.5.^13,21,37–39^ We found that EV pretreatment did not alter the intracellular fate of yeasts in BMDMs, as PL-fusion remained inhibited. These results suggest that EVs alone are insufficient to drive full macrophage activation required for an effective antifungal response against . In addition, virulence mechanisms triggered by live yeasts may overcome the partial activation induced by EVs. On the other hand, the increase in yeast phagocytosis by BMDC pretreated with EV_HcG‑217B_ is correlated with a higher fungal load after 3 h. This was not observed with EV_HcG‑184A_, indicating that EV_HcG‑217B_ compromises the fungicidal effect of BMDC and reinforces the fact that different strains can release EVs with distinct properties.

Fungal EVs have emerged as promising candidates for vaccine development with encouraging results in experimental models. For example, we have found that immunization with EVs conferred significant protection against murine candidiasis, even in immunosuppressed mice.^40^ Similarly, EVs from different fungal species have induced protective responses in larvae, reinforcing the idea that this strategy may be effective across multiple infection models.^25,40–44^ Based on our findings, it became evident that EVs are capable of modulating interactions with innate immune cells. However, the immunomodulatory profile elicited by these vesicles appears to have a limited capacity to drive full protective immunity. These observations suggest that, although EVs can influence innate immune pathways, their ability to trigger robust and effective adaptive responses remains constrained.

Fungal EVs can influence the disease outcome not only by modulating the host cell activity but also by carrying compounds that could impact the microenvironment and the fungus itself.^33,43,45^ For instance, enzymes carried by EVs could hydrolyze host molecules, helping the fungus to survive within the host.^18,46^ Earlier studies from our group demonstrated that EV_HcG‑217B_ transports catalase, an enzyme involved in fungal protection in environments with oxidative stress, such as the phagolysosome.^18^ Although the catalase activity was confirmed in both EVs, we did not find differences that could explain why EV_HcG‑217B_ and EV_HcG‑184A_ displayed distinct effects in BMDCs. Finally, we investigated whether EVs have growth-promoting properties, as previously described for EVs.^47^ Remarkably, both EVs enhanced the fungal growth of their respective strains in vitro. It is plausible that this property may be exploited by when facing the intracellular environment, adding the ability to grow faster and survive in an adverse host environment. Taken together, our data support the hypothesis that EVs act as virulence factors during the histoplasmosis development. These EVs modulate host cell responses, promote fungal growth, and may function as an immune evasion strategy employed by .

## Conclusion

 employs diverse and unique strategies to survive within macrophages. Here, we demonstrate that EVs may act as virulence factors, enabling the fungus to evade the microbicidal activity of BMDMs and BMDCs. Our findings show that EVs are differently perceived by these cells compared to EVs from other fungal pathogens such as and . EVs from do not play a major role in the ability of the fungus to tolerate the intracellular compartments of BMDMs. However, EVs from the strain G-217B increased the internalization of the fungi by these cells. Additionally, EV_HcG‑217B_ increased the fungal load within BMDCs, suggesting that EVs contribute to tolerance of the G-217B strain within phagocytes. This may provide an opportunity for to disseminate from the lungs to other organs via the host immune cells. Further research is needed to identify the specific molecules involved in EV recognition and their effects on host cells. This knowledge will not only advance our understanding of host–pathogen adaptation but may also be exploited for the development of novel therapeutic strategies against histoplasmosis.

## Materials and Methods

### Culture Conditions of Fungal Strains

 strains G-217B (ATCC 26032) (chemotype I) and G-184A (chemotype II) were grown in modified Ham’s F12 medium (Invitrogen, USA) at 37 °C in a rotatory shaker at 150 rpm, as described.^48^ yeasts (strain 11) were cultivated in liquid Sabouraud medium for 48 h at 30 °C with shaking (150 rpm).

### EV Isolation, Quantification, and Size Characterization

EVs were isolated from one-liter culture supernatants of strains (G-217B and G-184A) and (strain 11) as described.^49^ Overall, the fungal culture supernatant was harvested after 48 h by centrifugation at 4000*g* for 15 min at 4 °C (Beckman Avanti J-E, JA-14 rotor) and subsequent filtration through a 0.45 μm filter (Merck Millipore). The supernatant was concentrated about 20-fold using the Amicon ultrafiltration system (cutoff, 100 kDa, Millipore). EVs were obtained by centrifugation at 100,000*g* for 1 h at 4 °C (Beckman Optima LE-80K, 70 Ti rotor) and washed twice in 0.1 M PBS pH 7.4 at 100,000*g* for 1 h at 4 °C. EV suspensions were plated on Sabouraud agar to confirm their sterility before experimental use.

The quantification of EV was correlated to ergosterol content using the Amplex Red Cholesterol Assay Kit (Thermo Fisher Scientific). Total protein content in fungal EVs were assessed by colorimetric detection using Pierce BCA Protein Assay Kit (Thermo Fisher Scientific). For particle size measurement, NTA analysis was carried out using the NTA 3.0 software (Malvern Panalytical) as described by Reis et al.^50^

### Catalase Activity Assay

Fungal EVs were diluted in 0.05 M phosphate buffer (pH 7.0) to a final concentration of 5 μM of ergosterol. A final volume containing 25 μL of each EV suspension was added to a 96-well flat-bottom UV-transparent microtiter plate (Greiner Bio-One) and mixed with 25 μL of 20 mM H_2_O_2_ in 0.05 M phosphate buffer (pH 7.0). The enzymatic activity was determined by following the decrease in absorbance at 240 mm every 5 s for 5 min at 22 °C. The reaction mixture consisted of 50 mM phosphate buffer (pH 7.0) and 20 mM H_2_O_2_. Catalase bovine liver (50 U/mL), purchased from Sigma (catalogue no. C-10), was used as a positive control, while H_2_O_2_ (20 mM) alone served as the negative control.

### Growth Kinetics

Growth kinetics assays were performed using the Bioscreen C-Microbiological Growth Analyzer (Labsystems, Finland). strains (5 × 10^6^ cells/mL), G-217B and G-184A, were incubated with 5 μM of EVs or PBS (control) in Ham’s F12 for 1 h at 37 °C. Plates were incubated at 37 °C for approximately 4 days, with 600 nm absorbance readings recorded every hour, and values were plotted against time to obtain the growth curves for each individual condition. The statistical analysis was performed using OD measurements at 48 h.

### Mammalian Cell Culture

Macrophages and DCs were obtained from C57Bl/6 bone marrow progenitors through differentiation in vitro using 20% L929-conditioned medium (source of M-CSF) and 20 ng/mL of recombinant granulocyte-macrophage colony-stimulating factor, respectively.^51,52^ Briefly, bone marrow cells were cultured at a concentration of 1 × 10^6^/mL in Petri dishes containing 10 mL of RPMI 1640 medium (Invitrogen, Life Technologies) supplemented with 1% penicillin–streptomycin and 10% fetal bovine serum and the respective supplements described above (differentiation medium). For BMDMs, an additional 10 mL of fresh differentiation medium was added on the third day, and adherent cells were recovered after 7 days of differentiation for use in the experiments. For BMDCs, 10 mL of fresh differentiation medium was added to the culture on day 3 and replaced on the sixth and eighth days. Differentiation was conducted for 10 days, and nonadherent cells were harvested for use in the experiments. BMDMs and BMDCs were characterized by F4/80 (FITC) and CD11c (APC) expression, respectively, with at least 80% of cells positive for each marker after differentiation. After differentiation, EV-treated cells were cultured in RPMI/DMEM medium (1:1, v/v) containing 1% penicillin–streptomycin and 1% v/v Nutridoma-SP (Boehringer Mannheim Biochemical, Roche Applied Science, Indianapolis, IN, USA) at 37 °C and 5% CO_2_.

### Internalization of EVs by Host Cells

BMDM and BMDC were plated onto 24-well plates covered with sterile glass coverslips (3 × 10^5^ cells/well) and incubated with EVs from G-217B or G-184A strains (1 μM of sterol content) for different lengths of time (60 min and overnight) at 37 °C and 5% CO_2_ atmosphere. EVs used in these experiments were previously stained with 3 μM DiIC18 (Invitrogen) for 30 min at 37 °C. The labeled EV suspensions were washed three times with PBS by centrifugation at 100,000*g* for 1 h at 4 °C (Beckman Optima LE-80K, 70 Ti rotor). DiIC18 incubated with PBS under the same conditions was used as the negative control. After the incubation period, host cells were washed with PBS and fixed with 4% paraformaldehyde (PF) for 30 min at room temperature. The slides were then washed three times with PBS and mounted in 50% glycerol and 50 mM n-propyl gallate in PBS. The slides were visualized with an AxioVision 4.8 (Carl Zeiss International) microscope or the fluorescence confocal microscopy laser Zeiss Elyra PS.1 (Zeiss, Oberkochen, Alemanha).

### Cytotoxicity Assay

Cell viability was determined by the colorimetric MTT assay, quantification of LDH activity, and annexin/propidium iodide (PI) staining (Supporting Information). For MTT assay and LDH activity quantification, BMDM and BMDC (10^5^ cells/well) were plated onto 96-well plate and treated overnight with different concentrations of EVs from G-217B or G-184A strains (0.05, 0.5, and 5 μM of sterol content) or PBS (negative control). 1% Triton X-100 (Sigma-Aldrich, EUA) treatment was used as a cell death positive control for these assays. The MTT assay was performed by adding 50 μg of MTT stock solution (thiazolyl blue tetrazolium bromide; Sigma-Aldrich, USA) to each well. The plate was incubated for 4 h at 37 °C, and then formazan crystals were dissolved with 100 μL of isopropanol. The formazan solution resulting in the coloring was read by absorbance at 570 nm. The secreted amount of LDH was determined using the Cytotoxicity Detection Kit^PLUS^ (LDH) (Sigma-Aldrich, EUA), according to the manufacturer’s protocol.

### Nitric Oxide and Cytokine Production

NO, TNF-α, IL-12, IL-6, and IL-10 were quantified in culture supernatant harvested from BMDM or BMDC that were treated overnight with different concentrations of EVs from G-217B or G-184A strains (0.05, 0.5, and 5 μM of sterol content) or PBS (negative control) at 37 °C. As a positive control, cells were treated overnight with 500 ng/mL LPS (lipopolysaccharides from O55:B5; Sigma-Aldrich, St. Louis, MO, USA). The cytokine production was assessed using ELISA kits (BD Biosciences, USA). Nitrite quantification, as an indicator of NO production, was measured using the Griess reagent (Promega catalog no. G2930).

### Western Blot

BMDM (1 × 10^6^ cells/well) was treated overnight with EVs isolated from G-217B or G-184A strains (5 μM of sterol content) or PBS (negative control) at 37 °C. As a positive control, cells were treated overnight with 500 ng/mL LPS plus 10 ng/mL recombinant murine IFN-γ (PeproTech, Rocky Hill, NJ, USA). Western Blot samples were prepared by cell scraping in 50 μL of ice-cold RIPA lysis buffer (150 mM NaCl, 50 mM Tris-Cl pH 8.0, 0.25% sodium deoxycholate, 1 mM EDTA pH 7.5, and 1.0% Nonidet P-40). The samples were centrifuged, and the supernatants were collected. A total of 20 μg protein were mixed with laemmli sample buffer and heated at 100 °C for 5 min. Cellular extracts were subjected to SDS/PAGE, and the proteins were transferred to nitrocellulose membranes. The membranes were blocked with TBS-T (10 mM Tris pH 7.5, 100 mM NaCl, and 0.1% Tween 20), containing 3% (w/v) BSA. For protein expression, rabbit polyclonal antibodies anti–NOS2 (M19, Santa Cruz) and mouse monoclonal antibody anti–GAPDH (6C5, Santa Cruz) were diluted in blocking buffer and added to the membrane for incubation overnight at 4 °C. After washes, primary antibodies were detected using appropriate horseradish peroxidase-conjugated antibodies diluted in TBS-T, containing 5% (w/v) fat-free milk. The horseradish peroxidase was detected on blot adding a luminol-based enhanced chemiluminescent substrate (SuperSignal West Pico PLUS Chemiluminescent Substrate, Thermo Scientific).

### Arginase Activity

BMDM was treated overnight with different concentrations of EVs isolated from G-217B or G-184A strains (0.05, 0.5, and 5 μM of sterol content) or PBS (negative control) at 37 °C. As a positive control, cells were treated with IL-4 (100 ng/mL). Then, cells were lysed with 100 μL of 0.1% Triton X-100, and then mixed with 100 μL of 25 mM Tris–HCl solution at pH 7.4 and 10 μL of 10 mM MnSO4. Arginase was activated by heating the sample for 10 min at 56 °C. The arginase reaction was carried out by incubating 100 μL of sample with 100 μL of 0.1 M l-arginine at pH 9.7 at 37 °C for 1 h. The reaction was stopped with 800 μL of a solution containing H_2_SO_4_, H_3_PO_4_, and H_2_O (1:3:7, v/v/v). Urea production was revealed by the addition of 40 μL of 10% α-isonitrosopropiophenone dissolved in 100% methanol and heating at 100 °C for 30 min. The absorbance was determined in a spectrophotometer at a 540 nm wavelength. Urea concentration was extrapolated from a standard curve linear up to 60 μg of urea. One unit of enzyme activity is defined as the amount of enzyme that catalyzes the formation of 1 μmol of urea/min.

### Surface Markers Analysis in BM-Derived DCs

BMDC (1 × 10^6^ cells/well) was recovered from a 6-well plate after overnight incubation with EVs isolated from strains G-217B, G-184A, and strain 11 (5 μM of sterol content), PBS (negative control), or stimulation with 500 ng/mL LPS (positive control) at 37 °C. Cells were washed with PBS and blocked with antimouse CD16/CD32 (BD Pharmingen, clone: 2.4G2) for 30 min at 4 °C. Then, cells were incubated with anti-CD11c (clone: N418 eBioscience, Thermo Fisher Scientific), anti-CD86 (clone: GL-1 eBioscience, Thermo Fisher Scientific), anti-CD80 (clone: 16-10A1 eBioscience, Thermo Fisher Scientific), and anti-MHCII (clone: NIMR-4—eBioscience, Thermo Fisher Scientific) for 30 min at 4 °C. Data was acquired on FACScalibur (BD Biosciences, San Diego, CA, USA), and analyzed with FlowJo software (BD Biosciences).

### Yeast Phagocytosis and Killing

BMDC was plated onto a 24 well plate (3 × 10^5^ cells/well) and treated overnight with EVs isolated from G-217B or G-184A strains (5 μM of sterol content) or PBS (negative control) at 37 °C. To determine phagocytosis of G-217B and G-184A strains by BMDC, yeasts were previously stained with 40 μg/mL of NHS Rhodamine (Thermo Scientific, Rockford, lL, USA) for 1 h at 37 °C, washed three times with PBS, and then added to cells attached to a glass coverslip at a multiplicity of infection of 5:1 (fungi/BMDC). After 1 h of incubation, cells were washed three times with PBS and then counterstained with 0.5 mg/mL Uvitex 2B for 20 min at 4 °C to distinguish internalized from noninternalized yeasts. Then, cells were washed three times with PBS and fixed with a 2% PF solution in PBS. On microscopy analysis, 200 cells per group were counted to determine the percentage of phagocytosis (total number of cells containing yeast internalized per total of cells in field) and phagocytic index (average number of yeasts inside macrophage). For the yeast killing assay, EV-treated cells were infected with G-217B or G-184A at a multiplicity of infection of 5:1 (fungi/BMDC) for 1 h, and then nonphagocytosed fungi were removed through three washes in PBS. Cells were kept in culture for an additional 2 h in fresh medium. Then, cells were washed three times and lysed by adding 100 μL of sterile ice-cold distilled water. The fungal suspension was plated on Brain-heart infusion agar (BHI) supplemented with 5% sheep blood, and plates were incubated at 37 °C for 1 week. The killing activity was expressed as absolute numbers of CFU.

### Determining Phagosome-Lysosome Fusion

BMDM was plated onto 24-well plates covered with sterile glass coverslips (3 × 10^5^ cells/well) and incubated overnight with EVs from G-217B or G-184A strains (5 μM of sterol content) or PBS (negative control) at 37 °C. Next, live and heat inactivated (60 °C for 30 min) G-217B or G-184A yeasts prestained with Uvitex 2B were added to BMDM pretreated with EVs (isolated from the respective strain) at a multiplicity of infection of 2. After 1 h of incubation, cells were stained by 70 nM LysoTracker (Thermo Fisher Scientific) for 30 min at 37 °C. Then, cells were washed three times with PBS and fixed with a 2% PF solution in PBS. Images were acquired in a fluorescence microscope Carl Zeiss Microscopy, AxioVision 4.8 software (Zeiss, Alemanha). 200 cells per group were analyzed using ImageJ software (Wayne Rasband, National Institutes of Health, EUA) to determine the percentage of colocalization (yeasts stained with both LysoTracker and Uvitex 2B per total of yeasts stained with Uvitex).

### Statistical Analyses

Statistical analyses were performed using GraphPad Prism software, version 8.4.3. Student’s *t*-test was used in experiments with two groups, and one-way ANOVA in experiments performed with three or more groups. The posthoc test Bonferroni was used after ANOVA.

## Supplementary Material



## Notes

Experimental details of apoptosis assay, including methodology and data presentation of apoptosis in BMDM incubated with EV_HcG‑217B_ or EV_HcG‑184A_ (PDF)
