# Supplementary material for: Extracellular vesicles from Distinct Strains Modulate Phagocyte Function and Promote Fungal Persistence
Source: ACS Infect Dis. 2025 Jul 14;11(8):2342–56. doi: 10.1021/acsinfecdis.5c00378 (PMC12340971; doi:10.1021/acsinfecdis.5c00378)
Supplement: Supplementary file 1 [file id5c00378_si_001.pdf]

Supporting Information

Extracellular vesicles from distinct *Histoplasma capsulatum* strains modulate phagocyte function and promote fungal persistence

Taiane N. Souza[1,2,5], Alessandro F. Valdez[1], Ana Claudia G. Zimbres[1,3], Bianca A. G. Sena[1,4], Flavia C.G. Reis[4], Marcio L. Rodrigues[1,4,8], Daniel Zamith-Miranda[2,5], Allan J. Guimarães[6,7,8], Alessandra A. Filardy[1], Joshua D. Nosanchuk[2,5] and Leonardo Nimrichter[1,7,8,].*

1 Universidade Federal do Rio de Janeiro, Instituto de Microbiologia Paulo de Góes, Rio de Janeiro, Brazil;

2 Division of Infectious Diseases, Department of Medicine, Albert Einstein College of Medicine, Bronx, New York 10461, USA;

3 Centro de Desenvolvimento Tecnológico em Saúde (CDTS), Fundação Oswaldo Cruz, Rio de Janeiro, Brazil

4 Instituto Carlos Chagas, Fundação Oswaldo Cruz (Fiocruz), Curitiba, Brazil;

5 Department of Microbiology and Immunology, Albert Einstein College of Medicine, Bronx, New York 10461, USA;

6 Departamento de Microbiologia e Parasitologia – MIP, Universidade Federal Fluminense, Instituto Biomédico, Rio de Janeiro, Brazil.

7 Rede Micologia RJ – Fundação de Amparo à Pesquisa do Estado do Rio de Janeiro (FAPERJ), RJ, Brazil;

8 National Institute of Science and Technology (INCT) in Human Pathogenic Fungi, Brazil.

Corresponding Author

Leonardo Nimrichter - Instituto de Microbiologia Paulo de Góes, Centro de Ciências da Saúde (CCS), Universidade Federal do Rio de Janeiro (UFRJ), Rio de Janeiro, RJ, Brazil.

Email: nimrichter@micro.ufrj.br

METHOD

Apoptosis assay

Apoptosis was determined in BMDM (4 x 10^5 cells /well) overnight treated or left untreated with EV (5 μM of sterol content) isolated from strains G-217B e G-184A at 37 ˚C. Treatment with 15 mM saponin was used as a positive control in this assay. Cells were harvested from the plate using Accutase (Sigma-Aldrich, EUA), and stained with propidium iodide (PI) and FITC-labeled annexin-V according to the manufacturer's instructions (Sigma-Aldrich, EUA). Data was acquired

on FACScalibur (BD Biosciences, USA), and analyzed with FlowJo software (BD Biosciences, USA).

FIGURE

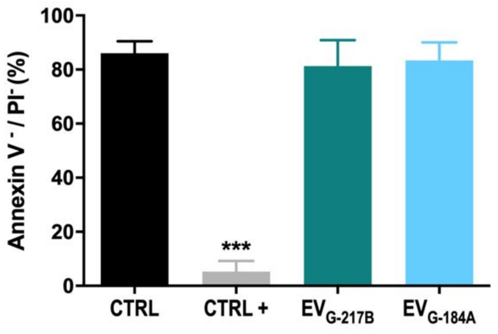

Figure S1. Apoptosis evaluation of BMDM incubated with $EV_{HcG\text{-}217B}$ or $EV_{HcG\text{-}184A}$. BMDM were stimulated overnight with 5μM of $EV_{HcG\text{-}217B}$ or $EV_{HcG\text{-}184A}$, and apoptosis was assessed by Annexin V/PI apoptosis assay. As positive control, BMDM were incubated with 15mM saponine. Graphs show means ± SD from 3 independent experiments. ***$p < 0.001$, one-way ANOVA comparison to untreated cells followed by the Bonferroni correction.
